# Effects of waste sources on performance of anaerobic co-digestion of complex organic wastes: taking food waste as an example

**DOI:** 10.1038/s41598-017-16068-z

**Published:** 2017-11-16

**Authors:** Xingang Lu, Wengang Jin, Shengrong Xue, Xiaojiao Wang

**Affiliations:** 1 School of Chemical Engineering, Northwest University, Xi’an, 710069 China; 2 Bio-resources Key Laboratory of Shaanxi Province, School of Biological Science and Engineering, Shaanxi Sci-Tech University, Hanzhong, 723001 China; 3 College of Agronomy, Northwest A&F University, Yangling, 712100 China

## Abstract

Few studies have addressed how to blend wastes for anaerobic co-digestion. This study investigated the effects of waste sources on anaerobic co-digestion (AcoD) performance, by varying the quality of food wastes (FWs) from six sources in Xi’an region, China that were individually co-digested with pre-treated corn straw and cattle manure. These effects were analysed in terms of their volatile solid (VS) ratios, C/N ratios, and the chemical composition of the FWs. The results indicated that the VS ratios were not suitable as a common mixture method because the VS ratios at which the best methane potentials occurred differed significantly among the six FW groups. The C/N ratios within a 17–24 range resulted in better methane potentials when the FWs were co-digested with other wastes. Synergistic effects were found among the carbohydrates, proteins, and lipids of the FWs; however, the optimum ratios of these components could not be determined. Thus, the C/N ratio is recommended as a mixture method when co-digesting FWs with other organic wastes in selected region.

## Introduction

The technology of anaerobic digestion (AD) has attracted a great deal of attention over the past decade because of its important role in protecting the environment, reducing the consumption of fossil fuels, and supplying clean energy^1^. Most previous studies of AD have focused on improving its methane production efficiency by optimizing the digestion process, and anaerobic co-digestion (AcoD) has proven to be particularly valuable in this regard^2,3^.

The benefits of AcoD lie in balancing the carbon/nitrogen (C/N) ratio and the nutrient contents of the given organic waste, increasing the pH buffering capacity, decreasing the ammonia toxicity and the accumulation of volatile fatty acids (VFAs), and improving the biochemical conditions for microbial growth^4,5^. More importantly, AcoD has been shown to significantly improve the profitability of the bioenergy industry by enabling the industry to more effectively commercialize its products. During the past five years, more than 500 studies on AcoD have been published. Most of these studies tested whether mixing different wastes could synergistically improve the methane potential, or they tried to identify the best waste mixing ratios during AcoD. To achieve these goals, different wastes were always mixed based on their volatile solid (VS) or total solid (TS) ratios^6–8^. However, this method is not optimal for obtaining suitable mixture ratios, because the sources of organic wastes are extremely extensive and they exhibit obvious variations in their nutrient levels. The same types of wastes also have very different chemical characteristics, even if they have similar VS or TS contents, which results in significantly different results in terms of the mixture ratios and the accompanying methane yield. For example, a mixing ratio of 1:3 (by volume) is optimal for the co-digestion of food waste (FW) and cattle manure (CM), and it resulted in a specific methane yield of 233 mL g^−1^ VS^9^. However, another study indicated that the optimum FM to CM ratio was 2:1 (based on VS), and it resulted in a methane yield of 388 mL g^−1^ VS^10^. Additional studies have demonstrated that the methane production rate can be enhanced by 0.67–5.5-fold when FW is co-digested with manure, in comparison with manure digestion alone^11,12^. Thus, determining the optimum waste mixing ratios remains a substantial problem.

No effective methods have been approved to solve this problem, except for some advances in exploring how to blend wastes. The optimization methods for achieving optimum mixture ratios include neural networks^13,14^, response surface methodologies^4,14^, the simplex-centroid mixture design and central composite design 8, and linear and adaptive linear programming^15,16^. The organic wastes used for AD are composed of carbohydrates, proteins, and lipids. Thus, several studies have shown that interactive effects among carbohydrates, proteins, and lipids occur during AcoD^17,18^. However, because of the complex composition of the wastes, studies on the nutrient levels are still limited; thus, the applicability of optimizing the mixture ratios of these components is still limited.

The aim of this study was to investigate how different sources of organic wastes affect AcoD performance and to determine in which case the synergistic effects among the organic waste components generally occur. Accordingly, we attempted to determine whether there is a common method for mixing wastes from various sources. FW accounts for a large proportion of municipal solid wastes in both developed and developing countries. Additionally, the chemical composition of FW always exhibits great variations, which results in methane yields ranging from 0 (with a failure due to acidification) to 518 mL g^−1^ VS (generally higher than other organic wastes)^19,20^. Thus, we selected FW as the primary substrate to co-digest with other wastes. The effects of the VS ratios, C/N ratios, and chemical components of six sources of FW, pre-treated corn straw (PCS), and CM on AcoD performance were analysed and discussed.

## Results and Discussion

### Chemical characteristics and AcoD performance of FWs, PCS, and CM

The primary characteristics of the FWs are shown in Table 1. The results indicate that the six FWs had extremely variable chemical compositions. The C/N ratio is an important indicator of a suitable digesting condition. The C/N ratios of the FWs ranged from 9.1 to 20.7, which partially overlap with the recommended C/N range of 15.0 to 30.0^21,22^. However, they differed slightly from those in previous studies, which ranged from 13.2 to 24.5^23^. Carbohydrates accounted for nearly one-half of the total chemical composition of the selected FWs, which is in accordance with most previous studies. However, the crude protein and lipid contents differed substantially among the six FWs, as reflected by their standard deviations of 3.91 and 6.16, respectively. Previous studies showed that the protein and lipid contents ranged from 12.7 to 18.5%^24,25^ and from 0.4 to 35.0%^26,27^, respectively. The variations in the chemical compositions of the FWs must be attributed to the variable ratios of meat, oil, fruit, and vegetables, and to different cooking methods. Pre-treated PCS had a higher C/N ratio (48) than the FWs and CM; therefore, it was suitable for adjusting the C/N ratio in the mixtures. The CM had a relatively balanced C/N ratio and generally high fibre content^24^, which made it suitable as the basic substrate; there was a fixed VS ratio in the mixture to prolong the degradable period and reduce the risk of acidification.

**Table 1:** Chemical characteristics of FWs, PCS, and CM. ^a^Not determined.

	FW1	FW2	FW3	FW4	FW5	FW6	PCS	CM	Inoculum
TS (%)	17.5	18.7	20.3	22.5	16.6	19.8	48.2	15.6	9.6
VS (%)	16.5	17.2	17.8	20.1	14.2	18.0	46.5	12.4	6.8
VS/TS ratio	94.4	91.9	87.7	89.3	85.5	90.9	96.5	79.3	70.8
C (% TS)	37.3	39.9	40.4	42.2	43.4	43.5	53.7	46.6	nd^a^
N (% TS)	4.1	3.3	3.0	2.7	2.4	2.1	1.1	1.9	nd
H (% TS)	6.3	7.9	8.8	6.6	7.5	7.1	5.6	5.3	nd
O (% TS)	24.4	26.9	29.2	20.6	24.3	25.5	42	30.2	nd
C/N	9.1	12.0	13.4	15.9	18.1	20.7	48.8	24.4	nd
Carbohydrate (% TS)	57.7	51.2	50.7	48.3	51.4	46.2	91.3	63.1	nd
Crude Protein (% TS)	23.9	20.5	18.3	15.3	14.6	12.2	5.2	13.5	nd
Crude Lipid (% TS)	12.8	20.2	18.7	25.7	19.5	32.5	-	2.7	nd
Ash	5.6	8.1	12.3	10.7	14.5	9.1	3.5	20.7	36.8

All of the FWs had relatively similar TMPs, which ranged from 589 to 686 mL g^−1^ VS, but they had extremely different SMPs, ranging from 95 to 463 mL g^−1^ VS (Table 2). In particular, the average SMP and biogasifiability of FW3, FW4, and FW6 were 3.4- and 3.3-fold higher, respectively, than those of FW1, FW2 and FW5. The pH value of the FW generally ranged from 3.9 to 5.2 as reported by previous research^28^, and when the FWs were mixed with inoculum (with a pH value of 7.16 in the present study), the pH of the inoculated mixtures increased (Table S1). However, reactors loading FW1, FW2 and FW5 became acidic quickly with low pH values (Table 2, S1), indicating the accumulation of VFAs, and consequently, methanation was inhibited during AD. The methane yields of the FWs ranged from 0 to 518 mL g^−1^ VS in previous reports, and this finding supports the results in the present study^19,20^. In short, although different FWs had relatively similar TMPs, their SMPs and biogasifiability differed substantially, which significantly affected their AD performance.

**Table 2:** Anaerobic digestion performance of individual FWs, PCS, and CM. ^a^Not determined.

	FW1	FW2	FW3	FW4	FW5	FW6	PCS	CM	Inoculum
TMP (mL g^−1^ VS)	589	617	642	677	686	674	507	548	nd^a^
SMP (mL g^−1^ VS)	95 ± 5.2	135 ± 11.3	422 ± 24.5	463 ± 30.3	163 ± 15.3	461 ± 20.6	298 ± 12.6	285 ± 20.4	53 ± 2.1
Biogasifiability (%)	16.1 ± 0.9	21.9 ± 1.8	65.7 ± 3.8	68.4 ± 4.5	23.7 ± 2.2	68.4 ± 3.1	58.8 ± 1.9	52.0 ± 3.0	nd
Averaged pH	4.24 ± 0.04	3.94 ± 0.03	6.87 ± 0.11	6.96 ± 0.07	4.79 ± 0.03	7.15 ± 0.09	7.32 ± 0.03	7.21 ± 0.01	7.34 ± 0.02

### Analysis of AcoD performance in terms of VS ratios

**Figure 1:**
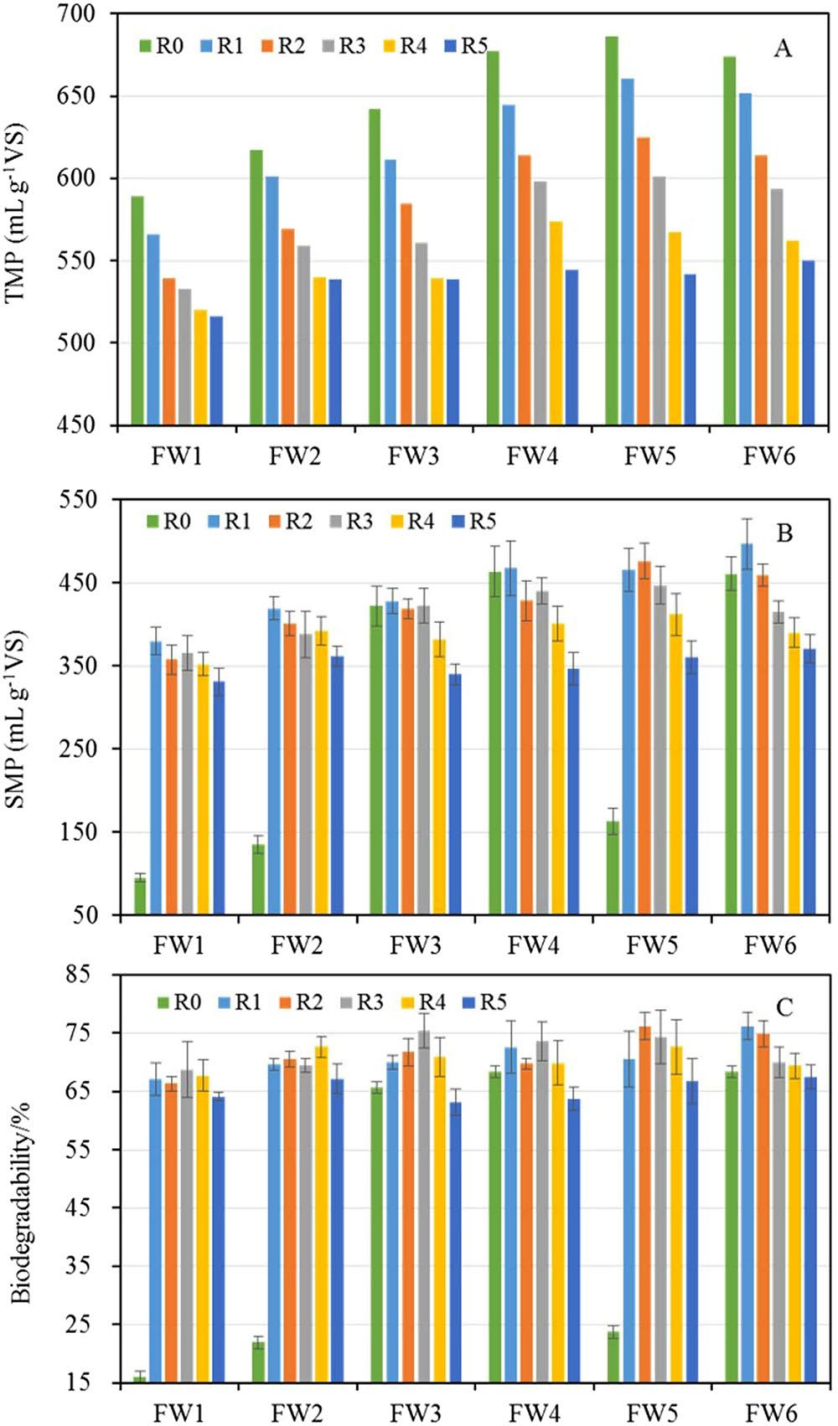
Anaerobic co-digestion performance of different mixtures. TMP, SMP, and biogasifiability of individual FWs (R0) and in the mixtures of FWs, PCS, and CM (R1 to R5) were separately presented in panel (A, B and C). The error bars are expressed as mean ± standard error (SE).

The TMPs of the mixtures containing FWs, PCS, and CM (Fig. 1A) were lower than the TMP of any single FW (Table 2), and the TMP tended to decrease as the ratio of the FW in the mixture decreased. This trend occurred because all of the FWs had higher TMPs and SMPs than the PCS and CM (Table 2). This result is also supported by a study in which the TMPs of FW, PCS, and CM were 724, 470, and 617 mL g^−1^ VS, respectively^29^. As shown in Fig. 1B, all six of the FWs (at all five VS ratios) successfully produced methane via co-digestion with PCS and CM, which confirmed that AcoD could reduce the risk of acidification that occurs when digesting FWs individually. The changes in pH values during the AcoD process shown in Table S1 supported this conclusion. The reasons were attributed to the fact that, for one thing, co-digestion diluted the inhibitory effects and improved nutrient balances as the substrate complexity increased^30^. For another thing, the inoculum and alkali inside of the PCS might neutralize the acids from FW and improve the buffer capacity of the mixture substrate.

However, the TMPs and SMPs of the six FWs differed greatly. Accordingly, the biogasifiability of the different FWs also showed extreme variations at all of the mixture ratios. We hypothesized that if there was no interactive effect in the FW, PCS, and CM mixture during AcoD, the variations in the SMPs and the biogasifiability among the six FWs should be consistent with the TMPs of the different mixture ratios. The biogasifiability fluctuations (Fig. 1C) suggested a clear interaction among the FW, PCS, and CM. In addition, because the biogasifiability of FW is higher than that of CS and CM, the biogasifiability of the mixtures should decrease as the proportion of FW in the mixture decreases. However, the maximum biogasifiability percentages in the FW1 to FW6 mixtures were 68.7, 72.6, 75.4, 73.6, 76.2, and 76.2%, which occurred in batches R3, R4, R3, R3, R2, and R1, respectively. Clearly, the biogasifiability in all these ratios was significantly (*P* < 0.05) higher than the other ratios in the group from the same FW source. Based on these results, we conclude that the sources of the FWs significantly affected the methane potential of mixed substrates during AcoD. This finding occurred because of the different chemical compositions of the FWs as well as the interactive effects among these organic wastes.

These interactive effects were found to be synergistic based on the following results. Synergism could be evidenced by an increase in the methane yield during the co-digestion of substrates by analysing the weighted average of the methane potentials from the individual substrates (the weighted SMP)^29^. If the value of the SMP minus the weighted SMP (mL g^−1^ VS) is greater than the value of the standard deviation of the SMP, it can be concluded that there was a synergistic effect during the AcoD. As a result of the inhibition that occurred for FW1, FW2, and FW5, only FW3, FW4, and FW6 were appropriate for analysing synergistic effects. As shown in Table 3, except for the R4 and R5 batches from FW4, the SMP values of the other mixtures were greater than their weighted SMP values, thereby demonstrating positive synergistic effects among the selected wastes. However, the values of the SMP minus the weighted SMP differed among the six FWs, and the highest values in the FW3, FW4, and FW6 groups were found in batches R4, R3, and R1, respectively.

**Table 3:** Synergistic effects as reflected by the positive differential (SMP minus the weighted SMP) and standard deviation.

	FW1	FW2	FW3	FW4	FW5	FW6
R1	nc^a^	nc	33.4^b^ (15.6)^c^	40.2 (33.1)	nc	70.8 (30.3)
R2	nc	nc	43.0 (11.8)	25.8 (24.2)	nc	57.8 (12.8)
R3	nc	nc	65.6 (20.8)	62.3 (16.4)	nc	38.1 (12.8)
R4	nc	nc	67.6 (21.1)	12.7 (21.2)	nc	37.7 (17.2)
R5	nc	nc	19.8 (12.7)	18.6 (19.6)	nc	43.0 (17.2)

^a^nc, not calculated because of an inhibitory effect when FW1, FW2, and FW5 were digested individually under anaerobic conditions. ^b^This value equals the averaged SMP from each mixture substrate minus the weighted SMP calculated by separate waste indicated in equation 4. ^c^This value is the standard deviation of the SMP.

The SMPs were mostly affected by the ratios of wastes with high methane potentials in the mixtures (the FWs had higher methane potentials than the PCS and CM), and, therefore, we could not determine whether there was a synergistic effect during AcoD. Compared with the SMPs, the biogasifiability and synergistic effects more suitably reflected the AcoD performance. According to the aforementioned results, the mixture ratios with the best performance differed greatly among the six FW groups. Thus, we conclude that the VS ratio is not suitable for use as a common method for blending organic wastes under AcoD when the sources vary. By comparing our results with those of previous reports, we determined that the mixture ratios of FWs and CM (or other wastes) that yielded the highest methane potentials differed significantly, which supports our conclusion^9,10^.

### Analysis of AcoD performance in terms of C/N ratios

With increasing C/N ratios, mixed substrates with various FWs all showed decreasing trends in their SMPs during AcoD. All of the data points in each FW group exhibited significant, linear relationships, with *R* ^2^ values of 0.855, 0.881, 0.902, 0.907, 0.934, and 0.918 for FW1 to FW6, respectively. This finding occurred because the FWs had lower C/N ratios, but higher methane potentials, than the PCS and CM (Tables 1 and 2). Thus, with the increased C/N ratios in the mixtures, the proportions of FWs decreased, resulting in the decline of SMPs from these mixed substrates. In a previous study, researchers conducted an anaerobic batch experiment under four mixing ratios of FW and rice husks with C/N ratios of 20.0, 25.0, 30.0, and 35.0, and the highest SMP was achieved using a feedstock mix with a C/N ratio of 20.0^31^. This study also demonstrated that the methane yield decreased as the FW proportion decreased, which supports the results shown in Fig. 1B.

**Figure 2:**
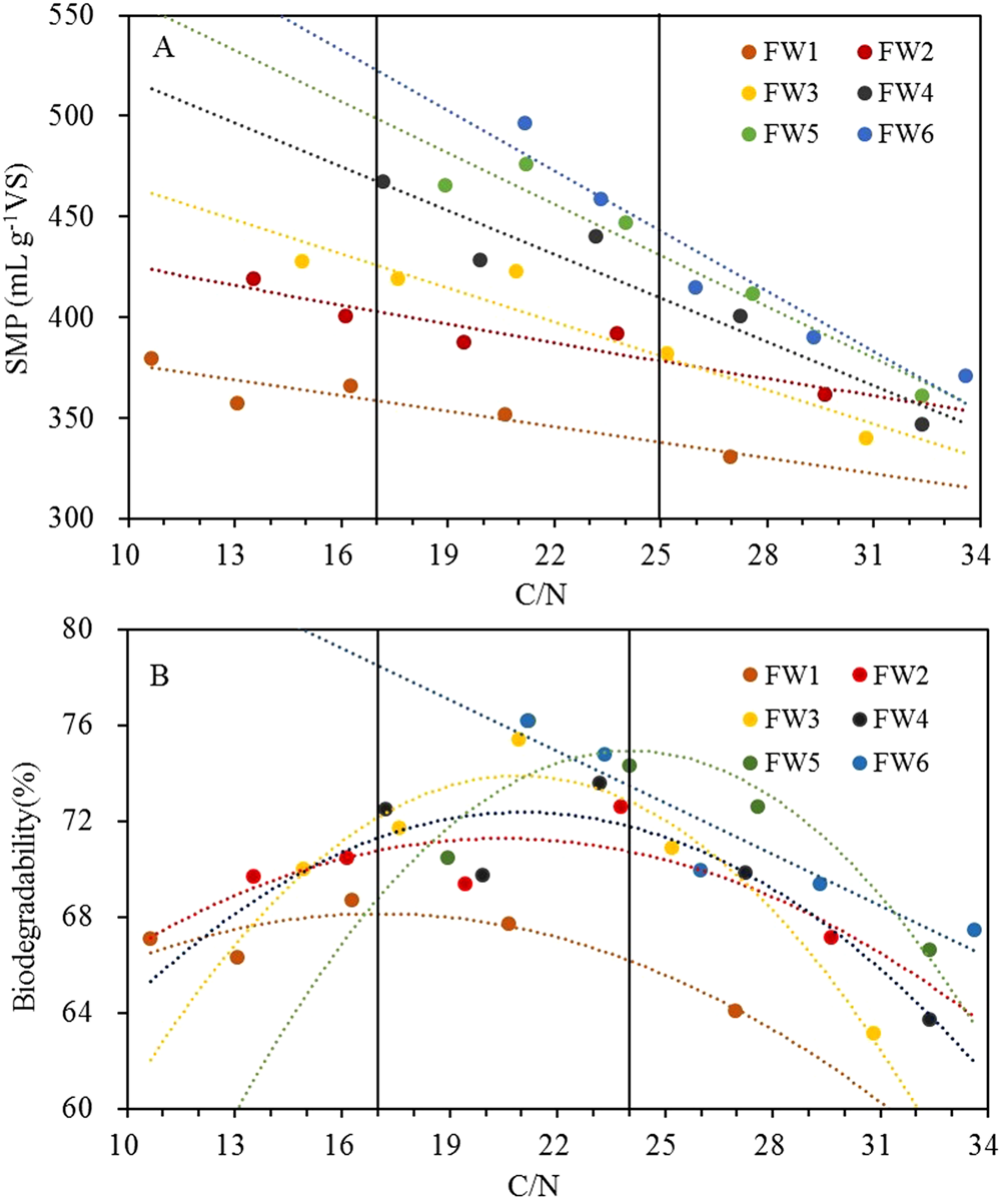
The relationships between C/N ratios and the SMP (**A**), as well as biogasifiability (**B**). The dots were the data of SMP or biogasifiability at different C/N ratios in different FW groups during AcoD. The dash line was used on behalf of the regression curve of data in each FW group.

The C/N ratios of the FWs ranged from 9.1 to 20.7 (Table 1). As shown in Fig. 2A, FWs with higher C/N ratios generally had higher SMPs, and this phenomenon was more obvious when the FWs accounted for a higher proportion of the mixed substrates. From previous reports, the optimal C/N ratios for AD range from 20.0 to 30.0^4,32^. In the present study, an analysis of all the data showed that high SMPs occurred in groups FW4, FW5, and FW6, whose C/N ratios ranged from 17.0 to 25.0 (Fig. 2A), which is partially in accordance with previous results. Thus, FWs with high C/N ratios made it much easier to achieve an appropriate C/N ratio, which resulted in high SMPs during AcoD.

The biogasifiability initially increased and then decreased as the C/N ratios increased in the groups from FW1 to FW5 (Fig. 2B). The benefits to AcoD in terms of balancing the C/N ratios have been demonstrated in many previous studies. Thus, the relationship between biogasifiability and the C/N ratio demonstrated the existence of a synergistic effect, which resulted from the adjusted C/N ratios when the FWs were co-digested with the PCS and CM. There was a linear relationship between biogasifiability and the C/N ratio for FW6 (*R* ^2^ = 0.885, *P* = 0.017), and the highest biogasifiability was obtained at a C/N ratio of 20.7. For FW1 to FW5, these relationships could be fitted by a quadratic curve, with *R* ^2^ values of 0.822, 0.542, 0.947, 0.831, and 0.858, respectively, and maximum biogasifiability occurred at C/N ratios of 17.0, 20.5, 20.9, 21.0, and 24.0, respectively (Table 4). Thus, high biogasifiability could be found in mixtures with C/N ratios ranging from 17.0 to 24.0.

**Table 4:** The C/N ratios in different mixtures of FW, PCS, and CM.

FW	Ratios of FW, PCS, and CM				
	R1: 80:0:20	R2: 65:15:20	R3 50:30:20	R4 35:45:20	R5 20:60:20
FW1	10.7	13.1	16.3	20.6	27.0
FW2	13.5	16.1	19.4	23.8	29.6
FW3	14.9	17.6	20.9	25.2	30.8
FW4	17.2	19.9	23.2	27.2	32.4
FW5	18.9	21.2	24.0	27.6	32.4
FW6	21.2	23.3	26.0	29.3	33.6

A subsequent data analysis of the SMP and biogasifiability revealed that different sources of FWs had extremely different AcoD performances because of the differences in their C/N ratios. For the FW1 to FW6 groups, their highest biogasifiability and SMP results were 68.7, 72.6, 75.4, 73.6, 76.2, and 76.2%, and 380, 419, 428, 468, 476, and 497 mL g^−1^ VS, respectively, which were accompanied by C/N ratios of 9.1, 12, 13.4, 15.9, 18.1, and 20.7, respectively (Table 4). Further analysis showed that FW4, FW5, and FW6 had a higher average C/N ratio (18.2) than FW1, FW2, and FW3 (11.5), which generally resulted in a higher average biogasifiability and SMPs of 74.5% and 480 mL g^−1^ VS for FW4, FW5, and FW6, compared with 71.1% and 408 mL g^−1^ VS for FW1, FW2, and FW3. In conclusion, FWs possessing a high C/N ratio, generally higher than 15.0, exhibited superior performance during AcoD with other organic wastes. Thus, when FWs are used for AcoD, the C/N ratio of the mixture should be adjusted to fall within a range from 17.0 to 24.0. This conclusion is supported by many AcoD studies in which the optimum C/N ratios of the FWs ranged from 20 to 21.7^27,31–33^. However, some reports obtained appropriate C/N ratios of 12.7^34^, 15.8^10^ and 15.0^25^, which are relatively lower than those in the present study. This finding occurred because the C/N ratios of the FWs were generally less than 20.0 in those studies, and the FWs were co-digested with substrates, such as pig manure, chicken manure, algae and sewage sludge, which had even lower C/N ratios. By comparing the results of the present study with those of previous reports, we could not determine which type of waste produced the highest methane potential when co-digested with FWs. However, according to our results, when FWs are typically and primarily used for AcoD, the C/N ratios are recommended in the range from 17.0 to 24.0.

### Analysis of AcoD performance in terms of chemical compositions

**Figure 3:**
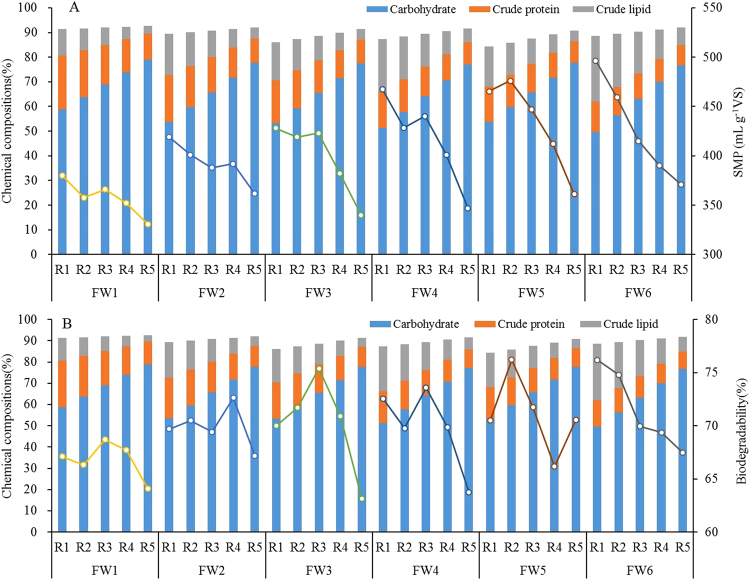
Chemical components of the six FW groups at different mixing ratios, and their related SMP (**A**) and biogasifiability (**B**) during AcoD. The columns presented the data of chemical components and the folding line presented the data of SMP or biogasifiability in each FW group.

As shown in Fig. 3A, the SMP results generally decreased with increasing carbohydrate contents and decreasing crude lipid contents. Thus, the carbohydrate content was negatively correlated with the SMP, while the crude lipid content was positively correlated with the SMP (Table 4). Theoretically, the methane potentials obtained from carbohydrates, proteins, and lipids are 415, 496, and 1,014 mL g^−1^ VS^35^, respectively. Thus, the fact that fewer FWs resulted in lower SMPs during AcoD could be clearly explained by the chemical compositions. The high lipid contents of the FWs easily resulted in the accumulation of long-chain fatty acids (LCFA), and they were accompanied by methanation inhibition^12,13^. All the mixtures of the six FW groups successfully produced methane, suggesting that there were synergistic effects among their chemical components. Previous studies showed that mixing a carbohydrate and/or protein source with lipids was a feasible option for reducing LCFA-mediated methanation inhibition^17,36^. However, the difference in the SMPs among the six FW groups could not simply be explained by any single chemical component. Even when different FW groups had similar carbohydrate, protein, or lipid contents, differences in their SMPs were as high as 95, 140, or 75 mLg^−1^ VS, respectively. Thus, the AcoD performance greatly depended on the synergy among the chemical components, which is further described in the following sections.

The carbohydrate and lipid contents of the FWs were statistically correlated with the biogasifiability according to all of the data (*P* < 0.001), but their correlation coefficients were as low as −0.593 and 0.648, respectively (Table 5). For each FW group, the correlation between biogasifiability and the carbohydrate or lipid content was not statistically significant. The maximum biogasifiability of the six FW groups occurred in batches R3, R4, R3, R3, R2, and R1, in which the carbohydrate contents were 68.9, 71.6, 65.4, 64.2, 59.7, and 49.6% TS, respectively, and the lipid contents were 6.9, 7.6, 9.9, 13.4, 13.2, and 26.5% TS, respectively (Fig. 3B). Thus, the wide range of carbohydrate and lipid contents made it impossible to conclude which individual chemical component resulted in maximum biogasifiability. Increased biogasifiability was related to an increased SMP in some of the mixtures. For example, in the FW5 group, batch R2 had a lower lipid content but higher biogasifiability than batch R1, which resulted in a higher SMP in batch R2. Similar results were also found for batches R3 and R4 of the FW2 group, batches R2 and R3 of the FW3 group, and batches R2 and R3 of the FW4 group. These results suggest that the increased biogasifiability could overcome the negative effect of a decrease in the lipid content of a mixture, thereby resulting in a high SMP. The increased carbohydrate contents may have enhanced the degradability of the lipids. The high biogasifiability and related high SMP again demonstrate the existence of synergistic effects among the chemical components.

**Table 5:** Correlation coefficients between chemical compositions and the SMP, as well as biogasifiability.

	Carbohydrate	Crude Protein	Crude Lipid
SMY	−0.813	0.162	0.858
*P* value	<0.001	0.393	<0.001
Biogasifiability	−0.593	0.098	0.648
*P* value	0.001	0.607	<0.001

Previous studies suggested that the biogasifiability and methane potential of a complex substrate are dependent on its chemical composition. For instance, the contents of biodegradable carbohydrates, proteins, and lipids can synergistically improve AD performance^23,37^. However, the way in which these synergistic effects occur among these chemical components was not clearly answered in the present study. As Table 3 indicates, the FW3, FW4, and FW6 groups had significant synergistic effects on the SMP, but their chemical compositions were complex and differed greatly. In addition, all of the mixtures in which the maximum SMP and biogasifiability occurred had different ratios of carbohydrate, protein, and lipid contents. Thus, the ratios at which these chemical components showed the greatest synergistic effects are still uncertain.

Although the present and previous studies showed that the lipid content of substrate mixtures positively influences the SMP^23,38^, the upper value of the lipid content was not determined conclusively. When the lipid content was higher than 30%, LCFAs strongly inhibited methanation^39^. Another study indicated that a lipid content that was 60% of the total VS content was the highest lipid content that did not inhibit AD^40^. In the present study, synergies between the substrates improved the AD rate and reduced the inhibitory effect of LCFAs, and, consequently, the lipid content could be as high as 66%^17^. However, we could not identify the conditions under which the synergistic effects occurred. Thus, although lipids played a dominant role in determining the SMP, synergistic effects among carbohydrates, proteins, and lipids should not be neglected when different organic wastes are used for AcoD. In conclusion, because of the unclear nature of the interactions among carbohydrates, proteins, and lipids, it is difficult to determine the optimum ratio between FWs and other wastes simply by assessing their chemical compositions.

## Conclusions

The chemical characteristics and AD performances of FWs from different sources varied greatly. The VS ratio was not suitable as a common method for blending wastes because the VS ratios at which the best methane potentials occurred differed significantly among FWs. When FWs are primarily used for AcoD, the C/N ratios of the mixture should be adjusted to 17.0 to 24.0, which will enable better methane potentials to be achieved. Synergistic effects among carbohydrates, proteins, and lipids played an important role during AcoD; however, their optimum ratios were not clearly identified. The C/N ratio is recommended as a mixture method for FWs under AcoD.

## Material and Methods

### Sources of substrates and inoculum

Raw food wastes (FWs) were obtained from six different restaurants in the area, and these wastes primarily contained meat, vegetables, rice and noodles. The collection of FWs was completed within two days, and the samples were all constantly kept at 0 °C before and after being transferred to the laboratory. Cattle manure (CM) was collected from a local livestock farm and Corn straw (CS) was obtained from a local villager. After crushed into particles using an electric grinder, with an average size of 2–3 mm, CS was first soaked with water to adjust its moisture content to 50% and then NaOH was added in an amount equivalent to 4% of the dry weight of the corn straw. The mixture was stirred once a day and the treatment lasted for 5 days. After pretreatment, the corn straw was taken from the plastic bucket and washed two times for a total of 3 min with distilled water to remove the alkali residue from the surface. The anaerobic sludge used as inoculum was collected from an anaerobic digester in a local village. The chemical characterization of each substrate and the inoculum used in this study are shown in Table 1. All the samples were collected in triplicate, and the averages of the three measurements are presented.

### Experimental design and set-up

Six FW samples were separately mixed with PCS and CM for AcoD. To reduce the risk of FW-mediated acidification, CM always accounted for 20% of the VS content in all of the mixtures. The VS ratios of the FW, PCS, and CM were then set to 80:0:20, 65:15:20, 50:30:20, 35:45:20, and 20:60:20. The PCS was used to adjust the C/N ratios of the mixed substrates. Anaerobic batch digestion tests were conducted in triplicate in 1-L filter bottles with a 600-mL working volume. The initial substrate-to-inoculum VS ratio was maintained at 1:2, and each mixture displayed an initial loading of 20 g VS/L (exclusive of the VS from the inoculum). For the control reactor, the same amount of inoculum was loaded and the rest of the working volume was filled with water. AcoD was conducted in triplicate at 35 °C for 35 days. Each reactor was flushed with N_2_ for 3 min to achieve an anaerobic condition, both initially and throughout the digestion process, and all of the reactors were shaken manually once per day before measuring the biogas yield.

### Analytical methods

TS and VS were analysed according to APHA Standard Methods^41^. Crude protein was estimated by multiplying the total Kjeldahl nitrogen by a factor of 6.25. Crude lipids were determined with a Soxhlet extractor using petroleum ether as the solvent^42^. The TS values of the carbohydrates were calculated by subtracting the crude protein, lipids, and ash^30^. An elemental analysis was conducted using an element analyser (Costech ECS 4024, Italy). The pH value of each mixture before and during the digestion process was measured with a pH metre (Leici PHS-25, Shanghai, China). The daily biogas volume was determined by measuring the displacement of water. The methane content was measured by a fast methane analyser. The SMP for each single substrate or mixture was presented as the total methane production of each reactor after subtracting the methane produced by the inoculum. The theoretical methane potential (TMP) was calculated according to the elemental composition, as shown in Eqs (1) and (2)^29^. Biogasifiability, which represents the AD efficiency, was used to indicate the performance of different FWs when they were co-digested with other wastes. This parameter was calculated using Eq. (3), in which SMP is the specific methane potential in a designated batch test.

1 $$\begin{array}{c}{{\rm{C}}}_{{\rm{n}}}{{\rm{H}}}_{{\rm{a}}}{{\rm{O}}}_{{\rm{b}}}{{\rm{N}}}_{{\rm{c}}}+({\rm{n}}-\frac{{\rm{a}}}{{\rm{4}}}-\frac{{\rm{b}}}{{\rm{2}}}+\frac{{\rm{3c}}}{{\rm{4}}}){{\rm{H}}}_{{\rm{2}}}{\rm{O}}\to (\frac{{\rm{n}}}{{\rm{2}}}+\frac{{\rm{a}}}{{\rm{8}}}-\frac{{\rm{b}}}{{\rm{4}}}-\frac{{\rm{3c}}}{{\rm{8}}}){{\rm{CH}}}_{{\rm{4}}}\\ \,\quad \quad \,\,\quad \quad +(\frac{{\rm{n}}}{2}-\frac{{\rm{a}}}{{\rm{8}}}+\frac{{\rm{b}}}{{\rm{4}}}+\frac{{\rm{3c}}}{{\rm{8}}}){{\rm{CO}}}_{2}+{{\rm{cNH}}}_{3}\end{array}$$

2 $${\rm{TMP}}({\rm{mL}}\,{{\rm{g}}}^{-{\rm{1}}}\,{\rm{VS}})=\frac{22.4\times 1000\times (\frac{{\rm{n}}}{{\rm{2}}}+\frac{{\rm{a}}}{{\rm{8}}}-\frac{{\rm{b}}}{{\rm{4}}}-\frac{{\rm{3c}}}{{\rm{8}}})}{{\rm{12n}}+{\rm{a}}+\mathrm{16b}+\mathrm{14c}}$$

3 $${\rm{Biogasifiability}}\,( \% )=\frac{{\rm{TMP}}}{{\rm{SMP}}}\times 100 \% $$

### Statistical analysis

All the values are expressed as the means ± standard error (SE). An ANOVA and a correlation analysis were performed with Minitab version 15.0 software.

## Electronic supplementary material

Dateset 1
