# Supplementary material for: Effects of waste sources on performance of anaerobic co-digestion of complex organic wastes: taking food waste as an example
Source: Sci Rep. 2017 Nov 16;7:15702. doi: 10.1038/s41598-017-16068-z (PMC5691039; doi:10.1038/s41598-017-16068-z)
Supplement: Supplementary file 1 — Dateset 1 [file 41598_2017_16068_MOESM1_ESM.doc]

Effects of waste sources on performance of anaerobic co-digestion of complex organic wastes: taking food waste as an example

Xingang Lu^1^*, Wengang Jin^2^*, Shengrong Xue^3^, Xiaojiao Wang^3#^

^1^School of Chemical Engineering, Northwest University, Xian, China 71069.

^2^Bio-resources Key Laboratory of Shaanxi Province, School of Biological Science and Engineering, Shaanxi Sci-Tech University, Hanzhong, China 723001.

^3^College of Agronomy, Northwest A&F University, Yangling, China 712100.

*These authors contributed equally to this work.

**^#^**Correspondence and requests for materials should be addressed to X.J.W. (email: w-xj@nwsuaf.edu.cn).

|  | Initial | D3 | D5 | D10 | D15 | D20 | D25 | D30 | Final | Average |
| --- | --- | --- | --- | --- | --- | --- | --- | --- | --- | --- |
| FW1 R0 | 6.34 | 5.06 | 4.52 | 4.05 | 3.61 | 3.89 | 3.56 | 3.62 | 3.55 | 4.24 |
| FW1 R1 | 6.49 | 5.34 | 5.15 | 5.88 | 7.07 | 7.11 | 7.31 | 7.46 | 7.38 | 6.57 |
| FW1 R2 | 6.64 | 5.54 | 5.19 | 6.49 | 7.16 | 7.23 | 7.21 | 7.35 | 7.41 | 6.69 |
| FW1 R3 | 6.78 | 5.71 | 5.29 | 6.32 | 7.04 | 7.18 | 7.24 | 7.41 | 7.49 | 6.72 |
| FW1 R4 | 6.91 | 6.04 | 5.83 | 6.71 | 6.74 | 7.19 | 7.29 | 7.33 | 7.31 | 6.82 |
| FW1 R5 | 7.04 | 6.27 | 6.08 | 6.42 | 6.97 | 7.29 | 7.42 | 7.49 | 7.47 | 6.94 |
| FW2 R0 | 6.22 | 5.24 | 4.15 | 3.22 | 3.47 | 3.25 | 3.27 | 3.15 | 3.31 | 3.92 |
| FW2 R1 | 6.51 | 5.41 | 5.98 | 6.12 | 6.45 | 7.16 | 7.35 | 7.28 | 7.24 | 6.61 |
| FW2 R2 | 6.57 | 5.56 | 5.24 | 6.07 | 6.87 | 7.09 | 7.25 | 7.37 | 7.31 | 6.59 |
| FW2 R3 | 6.64 | 6.14 | 5.75 | 6.38 | 7.15 | 7.32 | 7.22 | 7.39 | 7.45 | 6.83 |
| FW2 R4 | 6.81 | 6.08 | 6.15 | 6.52 | 7.08 | 7.29 | 7.45 | 7.52 | 7.49 | 6.93 |
| FW2 R5 | 7.05 | 6.23 | 6.17 | 6.46 | 7.14 | 7.24 | 7.32 | 7.32 | 7.38 | 6.92 |
| FW3 R0 | 6.72 | 6.01 | 5.69 | 6.63 | 7.19 | 7.22 | 7.41 | 7.31 | 7.44 | 6.85 |
| FW3 R1 | 6.87 | 5.94 | 5.71 | 6.54 | 7.09 | 7.31 | 7.57 | 7.49 | 7.53 | 6.89 |
| FW3 R2 | 6.69 | 6.22 | 5.93 | 6.57 | 7.11 | 7.27 | 7.48 | 7.51 | 7.55 | 6.93 |
| FW3 R3 | 6.88 | 6.21 | 6.09 | 6.74 | 7.04 | 7.29 | 7.42 | 7.54 | 7.48 | 6.97 |
| FW3 R4 | 6.99 | 6.48 | 6.32 | 6.59 | 7.15 | 7.32 | 7.51 | 7.42 | 7.38 | 7.02 |
| FW3 R5 | 7.08 | 6.32 | 6.41 | 6.75 | 7.32 | 7.41 | 7.41 | 7.35 | 7.44 | 7.05 |
| FW4 R0 | 6.64 | 5.42 | 6.29 | 6.84 | 7.25 | 7.42 | 7.34 | 7.45 | 7.59 | 6.91 |
| FW4 R1 | 6.89 | 5.61 | 6.12 | 7.04 | 7.21 | 7.34 | 7.44 | 7.41 | 7.48 | 6.95 |
| FW4 R2 | 6.84 | 5.71 | 5.82 | 6.87 | 7.28 | 7.24 | 7.33 | 7.38 | 7.45 | 6.88 |
| FW4 R3 | 6.91 | 6.14 | 6.28 | 7.12 | 7.31 | 7.38 | 7.32 | 7.41 | 7.44 | 7.03 |
| FW4 R4 | 7.05 | 6.04 | 6.48 | 7.01 | 7.19 | 7.34 | 7.39 | 7.41 | 7.49 | 7.04 |
| FW4 R5 | 7.01 | 6.24 | 6.32 | 6.87 | 7.26 | 7.15 | 7.32 | 7.29 | 7.42 | 6.99 |
| FW5 R0 | 6.47 | 5.3 | 4.92 | 4.29 | 4.48 | 4.42 | 4.42 | 4.49 | 4.36 | 4.79 |
| FW5 R1 | 6.65 | 5.24 | 5.25 | 6.04 | 7.15 | 7.21 | 7.28 | 7.41 | 7.52 | 6.63 |
| FW5 R2 | 6.74 | 6.24 | 5.57 | 6.38 | 7.05 | 7.26 | 7.31 | 7.24 | 7.36 | 6.79 |
| FW5 R3 | 6.84 | 5.78 | 6.09 | 6.98 | 7.23 | 7.34 | 7.4 | 7.31 | 7.35 | 6.92 |
| FW5 R4 | 6.86 | 6.06 | 5.79 | 6.38 | 6.99 | 7.28 | 7.32 | 7.26 | 7.31 | 6.81 |
| FW5 R5 | 6.90 | 6.28 | 6.19 | 6.88 | 7.15 | 7.24 | 7.31 | 7.21 | 7.26 | 6.94 |
| FW6 R0 | 6.65 | 5.98 | 6.44 | 7.22 | 7.36 | 7.41 | 7.55 | 7.68 | 7.64 | 7.11 |
| FW6 R1 | 6.90 | 6.12 | 5.82 | 6.48 | 7.02 | 7.25 | 7.36 | 7.44 | 7.46 | 6.87 |
| FW6 R2 | 6.93 | 6.24 | 5.61 | 6.78 | 7.15 | 7.28 | 7.24 | 7.31 | 7.35 | 6.88 |
| FW6 R3 | 6.84 | 5.78 | 6.05 | 6.62 | 7.21 | 7.32 | 7.35 | 7.31 | 7.34 | 6.87 |
| FW6 R4 | 7.04 | 6.14 | 5.89 | 6.54 | 7.05 | 7.24 | 7.18 | 7.26 | 7.31 | 6.85 |
| FW6 R5 | 6.96 | 6.32 | 6.01 | 6.42 | 6.98 | 7.11 | 7.18 | 7.24 | 7.29 | 6.83 |

**Table S1**. Changes of pH values during anaerobic digestion of individual FWs (R0) and anaerobic co-digestion of FWs, PCS and CM (R1 to R5).
